# Investigation of the Powder Aerosol Deposition Method Using Shadowgraph Imaging

**DOI:** 10.3390/ma14102502

**Published:** 2021-05-12

**Authors:** Philipp Glosse, Stefan Denneler, Oliver Stier, Ralf Moos

**Affiliations:** 1 Department of Functional Materials, University of Bayreuth, Universitätsstraße 30, 95440 Bayreuth, Germany; Functional.Materials@uni-bayreuth.de; 2 Siemens AG, Otto Hahn Ring 6, 81739 München, Germany; stefan.denneler@siemens.com; 3 Siemens AG, Siemensdamm 50, 13629 Berlin, Germany; oliver.stier@siemens.com

**Keywords:** aerosol deposition method, vacuum kinetic spray, vacuum cold spray, shadowgraph imaging, schlieren imaging, flow visualization

## Abstract

The powder aerosol deposition method (PAD) is a vacuum-based spray coating technology. It allows for production of highly dense coatings at room temperature, especially of brittle-breaking materials. This yields new options for coating substrate materials that even melt at low temperatures. The film formation mechanism is called room temperature impact consolidation (RTIC). The occurrence of this mechanism is strongly linked to the gas jet used in the process. The velocity and direction of the particles in the gas jet forming between the nozzle orifice and the substrate are the main factors influencing the quality of the coating. This dependency aimed to be elaborated with a measurement setup and coating experiments and is shown in this work. We investigated the gas jet formation using a shadow optical imaging system. Regions of different gas density are visualized by this technique. Several parameter sets, in particular gas flow rates and chamber pressures, were investigated. In addition, coatings were produced on glass substrates with the same parameters. As a coating material, the superconducting ceramic-like magnesium diboride (MgB_2_) was chosen. A correlation between shadow images and thickness profiles of the coatings shows how the gas jet formation affects the uniformity of thickness. Shadow optical images provide valuable information on the flight direction of the particles and allow validation of simulation results.

## 1. Introduction

The powder aerosol deposition method (PAD) is a spray coating process also known as aerosol deposition method (ADM), vacuum kinetic spraying (VKS), or vacuum cold spray (VCS). Typically, ceramic powders are accelerated into a vacuum to form highly dense films on various substrate materials at room temperature [1,2].

In the present work, shadow-optical images of the gas jet formed during PAD are correlated with coatings produced under the same process conditions. This is expected to provide further insights into the influence of the gas dynamics on the layer formation. This study, therefore, provides an experimental-based insight on the influence of the jet formation during the PAD process.

### 1.1. Powder Aerosol Deposition Method

**Figure 1:**
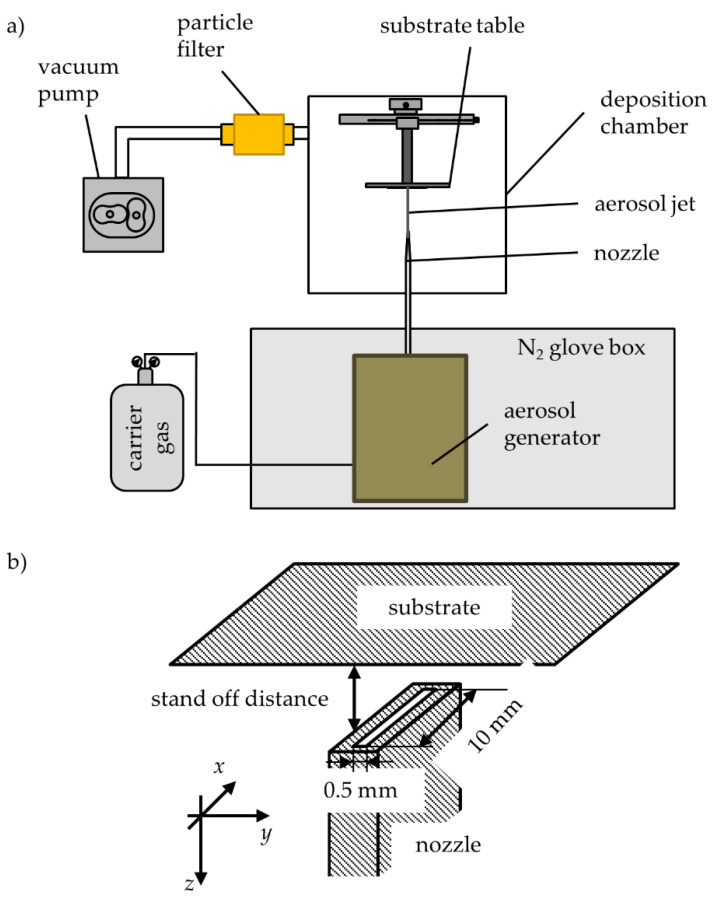
(**a**) Schematic drawing of an aerosol deposition device including deposition chamber, vacuum pump, and aerosol generating unit; (**b**) sketch of the nozzle-substrate-positioning.

The feasibility of the preparation of dense ceramic, metallic, and intermetallic thick films by the PAD has been shown in several works [1,2]. MgB_2_ films have been produced using this method [3,4,5,6]. The mechanism of the layer formation is described as room temperature impact consolidation (RTIC) [7,8]. The PAD is a powder-based room-temperature raw-vacuum process. Particles of the powder are aerosolized in a carrier gas by an aerosol generator. The aerosol is accelerated through a nozzle into a vacuum chamber and directed to a substrate such that the particles collide with the latter. The particles break and form a well-adherent layer through the reconsolidation of the particle fragments on the substrate surface. A sketch of the PAD setup is shown in Figure 1a. Figure 1b shows a close-up of the nozzle/substrate setup with the coordinate system used in this work.

During deposition, the substrates moves in positive or negative *y*-directions. The number of scans has significant effect on the film thickness [6]. Other parameters, such as the pressure ratio (stagnation pressure to backpressure), total carrier gas flow, and the particle size distribution of the powders, also need to be controlled for the successful production of film coatings by PAD.

### 1.2. Material

Superconductors are materials characterized by properties such as the disappearance of electrical resistance, the occurrence of ideal diamagnetic behavior, or the formation of quantized magnetic flux tubes [9]. Magnesium diboride (MgB_2_) is a promising superconducting material for films, filaments, or wires, which can be prepared by physical vapor deposition (PVD) and powder-in-tube (PIT) techniques. Both methods have certain disadvantages, such as the need for a particularly well-adjusted atmosphere in PVD, and a low filling factor for the PIT method. These processes run at high temperatures and involve expensive equipment [3]. Compared to high temperature superconductors like the rare earth barium copper oxides (ReBCO), the superconductivity of MgB_2_ is less anisotropic. Thus, there is no need for highly aligned crystal orientations. The low critical temperature of 39 K allows cooling with cryogenics but does not require liquid helium for use as superconductors in electrical devices. Generator coils, transformers, fault current limiters, and some more potential applications are overviewed in an article by Tomsic et al. [10].

The PAD is a promising manufacturing technology for thick-films (several µm), with a potential to overcome the high cost-value ratio of prior art production methods.

### 1.3. Particle Drag

Properties such as crystallite size, microstrain, and conductivities of coatings prepared from certain materials with PAD can be modified by thermal post-treatment [11], which can have a positive influence on their usability. However, there are various aspects thought to be influencing the deposition, like the powder pre-treatment [12] or aerosol generation [6]. Furthermore, the gas flow carrying the particles is considered the key factor for proper film formation. Hence, the velocity (*v_g_*), density (*ρ_g_*), pressure (*p_g_*) and temperature (*T_g_*) of the gas should significantly impact the deposition, since they influence the particle drag. The drag force (*F_D_*) is described by Equation (1) [13]:

(1) $$F_{D}=\frac{1}{2}C_{D}A_{p}\rho_{g}{\left(v_{g}-v_{p}\right)}^{2}$$

where *A_p_* represents the cross-sectional area of the particle, and *C_D_* the drag coefficient. The particle Reynolds number (*Re_p_*) and the Mach number (*Ma_p_*) influence the drag coefficient (*C_D_*). They can be calculated by Equations (2) and (3), respectively [14]. For their calculation, the dynamic viscosity (*µ_g_*), the particle diameter (*D*), the specific gas constant (*R*), and the gas temperature (*T_g_*) are required.

(2) $${Re}_{p}=\frac{\rho_{g}\left|v_{g}-v_{p}\right|D}{{µ}_{g}}$$

(3) $${Ma}_{p}=\frac{\left|v_{g}-v_{p}\right|}{\sqrt{{\gamma RT}_{g}}}$$

Equations (1)–(3) show that gas density and velocity are very important for a coating process that is driven by kinetic energy of the particles to be deposited. Good knowledge about the distribution of these properties within the two-phase flow is therefore crucial to optimize the PAD process.

**Figure 2:**
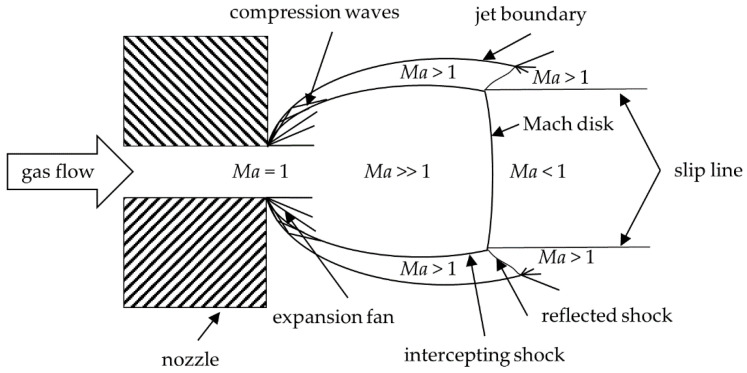
Scheme of a free gas expansion, modified after [13,24]. The gas flow expands in the low-pressure region downstream of the nozzle.

First finite element [15] and molecular dynamic simulations [8,16] that investigate the particle fracture behavior, provide hints on the powder pre-treatment and the particle velocities to be applied for successful PAD coatings. This indicates that a detailed knowledge on the influence of gas dynamics on spray coating processes can be beneficial. It has been investigated in several studies [17,18]. Katanoda et al. and Zabihi Yeganeh et al. presented interesting simulation-based work for PAD [19,20,21]. In the field of cold spraying, which is related to PAD, gas dynamic effects have been discussed by Pattison et al., Samareh et al., and Buhl et al. [13,14,22]. Moreover, Viscusi et al. reported on interesting results on the long-term stability of cold spray depositions based on numerical modeling of the gas flow regime and experiments [23]. According to [20], a basic description of the gas flow expanding into the vacuum can be described as in Crist et al. [24]. Such an expansion is schematically depicted in Figure 2.

The driving force to generate coatings by PAD is provided by the gas jet. Due to the large pressure ratio between the nozzle inlet pressure and the chamber (back)pressure, the gas jet is supersonic, and strongly underexpanded. The gas flow expands into a chamber with a very low backpressure. Thus, expansion waves form at the nozzle exit. These waves interact with the ambient pressure and are reflected at the jet boundary. The reflected waves are called compression waves [24]. These waves are reflected inside the jet, and an intercepting shock is formed. Between jet boundary and intercepting shock, the jet is supersonic. Somewhat downstream of the nozzle outlet, a Mach disk parallel to the nozzle exit plane forms. Downstream of the Mach disk, the flow is theoretically subsonic. The intercepting shock and the Mach disk interact and thereby form a reflected shock with continued supersonic flow behind it. The position of the shocks is important for the particle trajectories and the particle velocity. Further details of the situation of the jet behind the nozzle can be obtained from [20,24].

**Figure 3:**
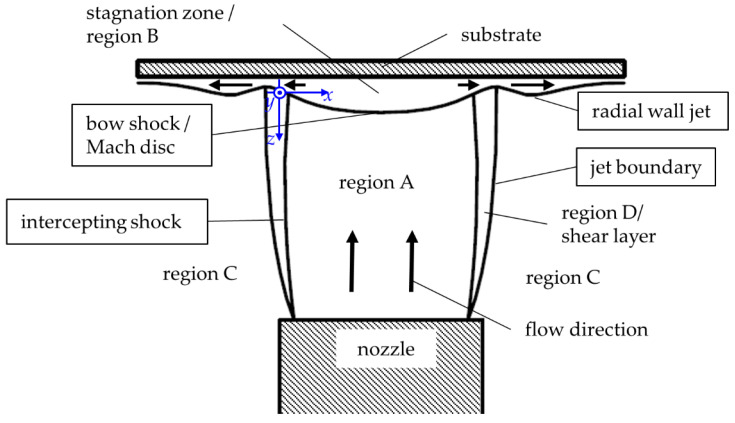
Scheme of the gas expansion between nozzle and substrate, including the different flow regions (region A to D) of the flow pattern, that are bounded by the shock waves (Mach disk, intercepting shock, jet boundary, radial wall jet). Modified from [13,24].

Different from Figure 2, where a free expanding flow is considered, Figure 3 depicts the gas flow between the nozzle and a substrate. The gas flow leaving the nozzle is supersonic in region A. Gas molecules of the jet, interacting with the substrate, change their energy and momentum. Infinitesimal pressure waves transfer these changes to outer regions of the flow. Since pressure waves can neither move up nor downstream, a shock wave forms. This shockwave is called bow shock. The flow downstream of the bow shock is subsonic (region B), and the flow transforms to an outward radial deflection. If the thickness of the bow shock is in the order of the mean free path of the gas molecules, the bow shock does not affect the particles. Between the bow shock and the substrate, a stagnation zone forms where the gas flow has a lower velocity and a higher density. There, the gas flow can even be recirculating [13]. The shear layer (region D) forms between the outermost jet boundary and the intercepting shock. In the boundary layer, expansion waves directed outwards (see expansion fan in Figure 2) are reflected as compression waves (marked in Figure 2) by the ambient pressure [13,25].

In the following, the gas flow without particles is explained using the gas density distribution, as visualized by a shadowgraph method like in [13,14]. The flow images are correlated with thickness profiles of the as-deposited films. In this work, the coating film quality is assessed by the thickness profile in the *x*-direction of the slit nozzle.

### 1.4. Shadow Optical Imaging

The shadowgraph method is an optical measurement method based on the varying light refraction in media of non-uniform density. It was already described by Dvořák in 1880 [26].

In its most simple assembly, light rays emitted from a point source pass the media under investigation and are collected on a screen. The light rays refract in different angles depending on the gas density. The intensity distribution of the transmitted light differs from the case of a homogeneous medium. The influence of the density (*ρ*) on the refractive index (*n*) is described approximately by Equation (4),

*n* − 1 = (*n*_0_ − 1) (*ρ*/*ρ*_0_), (4)

where *n*_0_ and *ρ*_0_ refer to a reference temperature and pressure [27]. The change of light intensity (Δ*I*) is proportional to the cross-sectional Laplacian of the refractive index (cf. Equations (5) and (6)) [27]. The light intensity that is integrated along the path from the light source to the screen (or camera), perpendicular to the flow direction, is represented by *I*.

(5) $$\frac{\Delta I}{I}=-\frac{1}{n_{0}}\overset{L}{\underset{0}{\int }}\left(\frac{\partial^{2}n}{\partial x^{2}}+\frac{\partial^{2}n}{\partial z^{2}}\right)dy$$

(6) $$\frac{\Delta I}{I}=-\frac{1}{n_{0}}\overset{L}{\underset{0}{\int }}\left(\frac{\partial^{2}n}{\partial y^{2}}+\frac{\partial^{2}n}{\partial z^{2}}\right)dx$$

The observed volume segment is described by the coordinates *x*, *y*, and *z*. Depending on whether the wide or the narrow side of the nozzle opening is observed, the illuminated image area is either the *x*-*z* or the *y*-*z* plane. The depth of each volume element thus extends in either the *y* or *x* direction.

**Figure 4:**
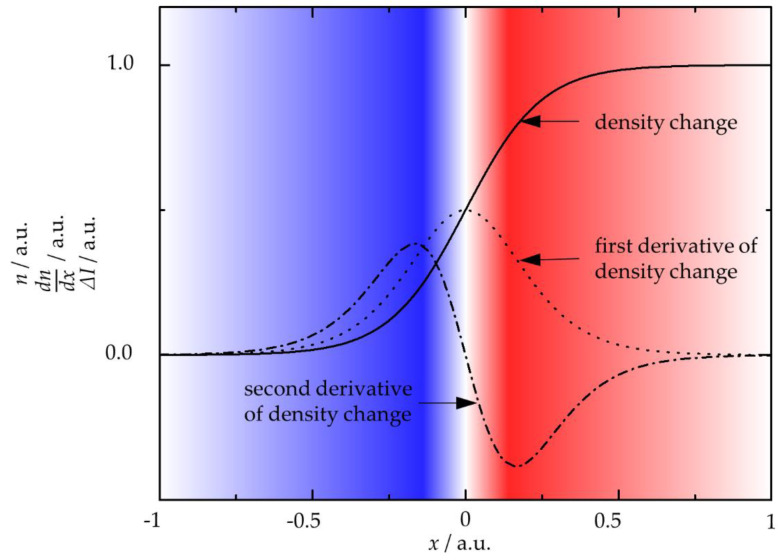
Example of the distribution of refractive index, *n* (density change), its first derivative, *dn*/*dx*, and the intensity change, Δ*I*, over *x*; the white-red-blue-transition highlights the Δ*I*(*x*).

In Figure 4, the distribution of the refractive index (*n*) and the diffraction gradient ($\frac{\partial n}{\partial x}$) are given in a schematic representation.

According to Equations (5) and (6), the change in the light intensity depends on the second derivative of the refractive index. As the refractive index is related to the gas density, the change in intensity (Δ*I*) depends on the gas density (*ρ*). A light beam crossing a region with a non-zero gas density gradient (i.e., with a refractive index gradient) generates an image where the boundary between the two different gas density regions is highlighted. The border appears as a dark-bright-transition. Image processing may convert those to color-images. In the neutral area, the second derivative is zero, and the blue-white-red transition marks the position of the density gradient (cf. Figure 4). The red colored side of the density gradient indicates a higher density than in zones framed in blue.

## 2. Materials and Methods

### 2.1. Shadow Optical Experiments

**Figure 5:**
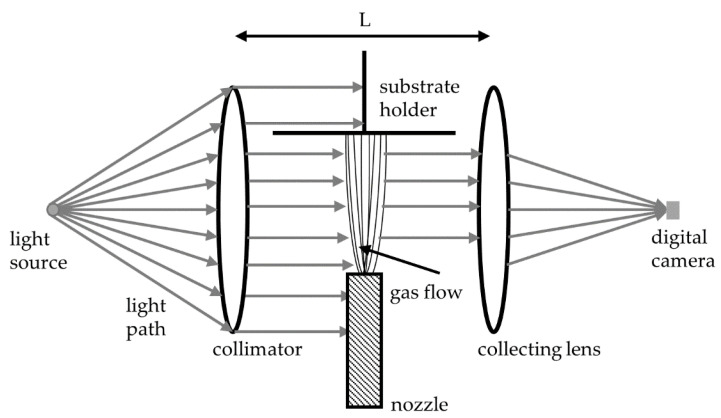
Scheme of a shadow imaging system consisting of a light source, collimating and collecting lens, and a camera system adjusted around the nozzle-substrate-setup.

The shadowgraph system used in this work consists of a white light source, a collimator lens, a collecting lens, and a digital camera system. The white light is collimated, refracted in the gas flow, and mapped to the camera chip (Figure 5).

The influence of the gas density distribution on the film formation is investigated in two experimental steps. Different parameter sets were investigated with the shadow imaging system without particle feeding. Corresponding coatings were produced by the PAD using a powder-laden gas, with otherwise identical flow parameters. The gas flow rate and the chamber (back)pressure were the main varied parameters. Nitrogen served as carrier gas at flow rates of 10, 15, and 20 standard liters per minute (slm). One slm is equivalent to 0.075 kg/h here. All gas flow rates were applied at the minimal possible (back)pressure in the vacuum chamber. In addition, 10 mbar, 15 mbar, and 20 mbar chamber pressures were set at all gas flow rates in order to obtain an experimental matrix. The stand-off distance between the substrate and nozzle were kept constant at 10 mm. The same slit nozzle was used in all experiments.

### 2.2. PAD Eexperiments

For the MgB_2_ coatings on glass substrates, a powder supplied by Alfa Aesar and jet milled down to a particle size of *d*_90_ < 5.4 µm by DEC, Ecublens/Lausanne Switzerland was used. The parameter sets used for coating preparation are given in the section “shadow optical experiments” (also cf. Table 1), and so is the nozzle used. The aerosol was generated with a RBG 1000 aerosol generator from Palas GmbH, Karlsruhe, Germany. Hanft et al. have already shown that this generator provides a well-defined aerosol that is suitable for PAD [6]. The particle concentration was maintained constant for all prepared films by adjusting the powder feed velocity of the aerosol generator. To provide the same powder mass of approximately 0.75 g for each single deposition process, the number of scans was adjusted accordingly. At a gas flow rate of 10 slm and a chamber pressure of 20 mbar, no coating was produced.

To compare the different deposited films, their thickness profiles were measured with the optical metrology system Microspy CF (sensor: AF16) from the FRT GmbH (Bergisch Gladbach, Germany).

## 3. Results

### 3.1. Gas Flow Visualization

**Figure 6:**
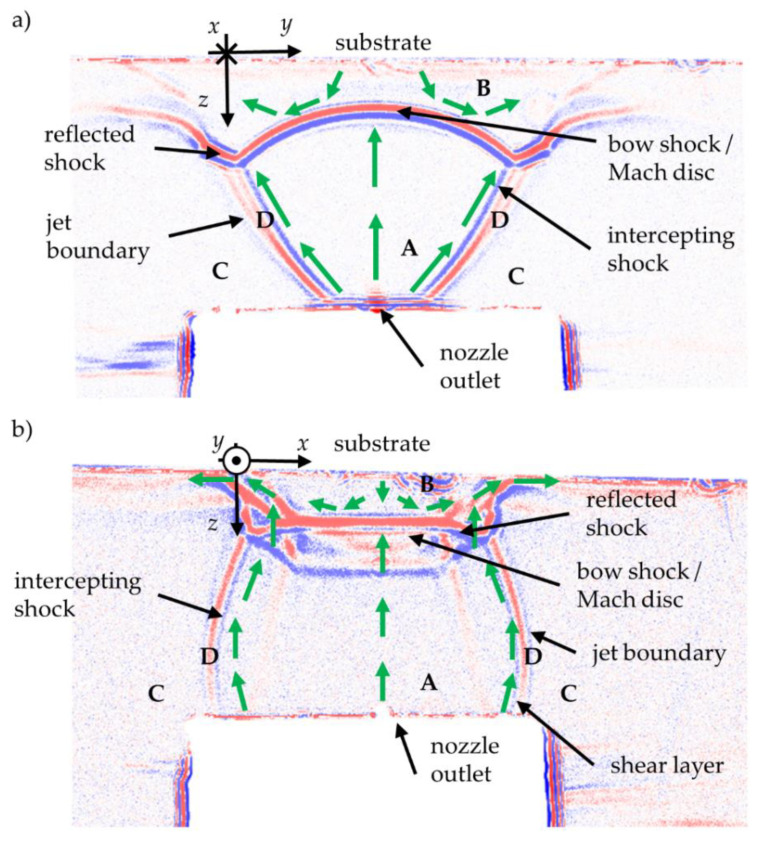
Shadow images of a PAD gas jet expansion, (**a**) *y*-*z* plane (slit of the nozzle), and (**b**) *x*-*z*-plane (width of the nozzle) with the different density regions A–D.

The (one-phase) gas flow was visualized by the shadow optical imaging system. The images are interpreted in the following, based on a simulation of Katanoda et al. and Zabihi Yeganeh et al. [20,21]. The shadow images in Figure 6 show the density distribution in the *y*-*z*-plane (0.5 mm small slit opening of the nozzle (a); and in the *x*-*z*-plane (10 mm large width of the nozzle (b). The expansion region in the shadow image of Figure 6a has the shape of a bell.

The jet shape fits qualitatively to the simulated ones of [20,21]. The measurement in Figure 6b shows a different jet shape. The jet looks slightly compressed in the *x*-direction. The different jet shape results from the high aspect ratio (*x*/*y* = 20) of the nozzle used. In both images, four clearly different density zones can be seen. The pressure in region C needs to be the same as the chamber pressure, i.e., the density is very low. The density in region D is higher than in regions A and C. In region B, the density is highest, as visualized by the boundary colors between the different regions. Red is thought to be on the side with a high density, and blue on the side with lower density. Regions A and B are divided by the bow shock, and region D has to be the shear layer, with the intercepting shock on the inner face and the jet boundary on the outer face [13,24].

Based on the mentioned simulations from references [20,21], gas streamlines are expected, as indicated in the shadow images by green arrows. The streamlines change their direction as they approach the substrate. In region B, a flow direction reversal towards the nozzle may occur. The shadow images do agree qualitatively with the simulations of [20,21].

A Mach-1-surface is attached to the nozzle exit boundary (transonic flow in nozzle orifice) while *Ma* > 1 directly upstream of the bow shock. Downstream the bow shock, the gas returns to subsonic flow, *Ma* < 1.

**Figure 7:**
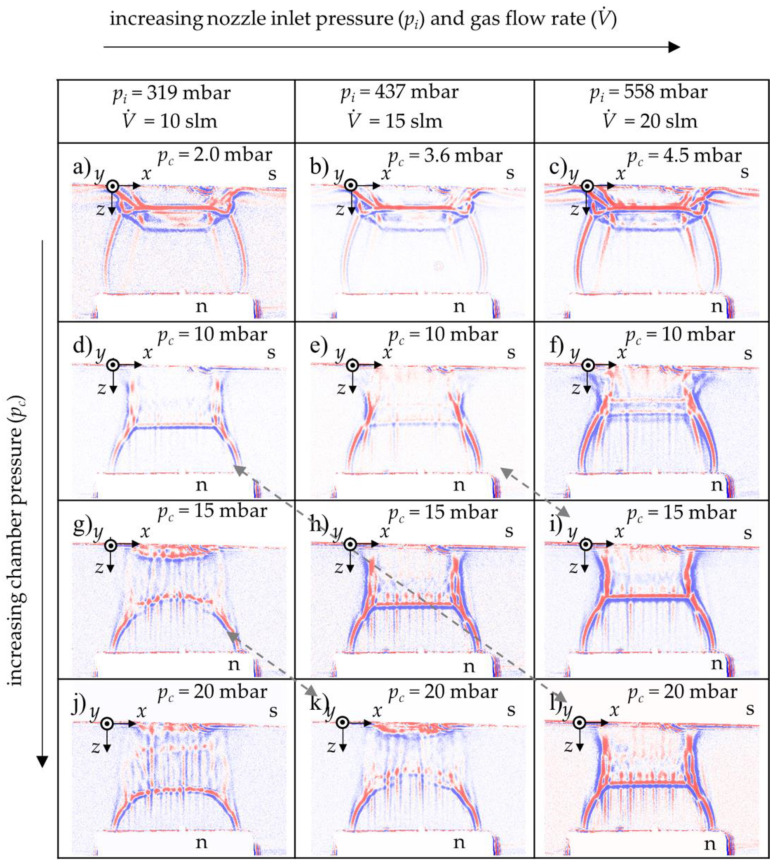
Expansion of carrier gas between the slit nozzle and substrate in *x*-*z*-plane (s: substrate, n: nozzle) of different parameter sets (gas flow rates and chamber pressures (**a**–**l**)); grey dashed arrows highlight qualitatively equal shadow images at different PAD parameter sets.

In Figure 7, the flow shape of the gas expansion in the *x*-*z*-plane is shown for all chamber pressures and carrier gas flow rates. A bow shock can be observed in all images. It is well visible that higher chamber pressures push the bow shock upstream, towards the nozzle exit. An increase in gas flow rate counteracts this, as explained below. The bow shock is formed with curved shape at lower flow rates and higher chamber pressures (cf. Figure 7g,j,k). Given the curved red-blue transition region upstream of the substrate and the high chamber pressure, the gas density upstream of the substrate should be higher than in the other shadow images.

For preparation of PAD films, usually the minimum chamber pressure possible is applied (Figure 7a–c). Here, the bow shock is very close to the substrate. In close vicinity of the substrate, some density field lines are seen to be diverted laterally. This indicates that the gas flows in *x*-direction. For higher chamber pressures, this effect occurs as well, but its detection is prevented by the trailing edge swirls at the substrate boundaries.

The dashed bidirectional arrows link parameter sets to each other which create similar gas expansion shapes, i.e., similar pressure ratios *p*_i_/*p*_c_ between the aerosol generator stagnation chamber (*p*_i_) and the vacuum chamber backpressure (*p*_c_). It may be assumed that such connected parameter sets lead to similar film properties. If this was true, PAD could advantageously be operated in a larger process window than before. Materials that could be deposited at a low gas flow rate only might be processed at higher gas flow rates in the future, restoring the optimal pressure ratio (*p*_i_/*p*_c_) by adjusting the chamber pressure. As a result, this would offer the opportunity to use different types of (commercially available) aerosol generators with improved dispersion quality, i.e., higher separation efficiency, and thus, promising higher deposition rates. This hypothesis still has to be confirmed by the PAD experiments (Section 3.2), since shadow images show a qualitative distribution of the density zones only.

### 3.2. As-Deposited Films Characteristics

**Figure 8:**
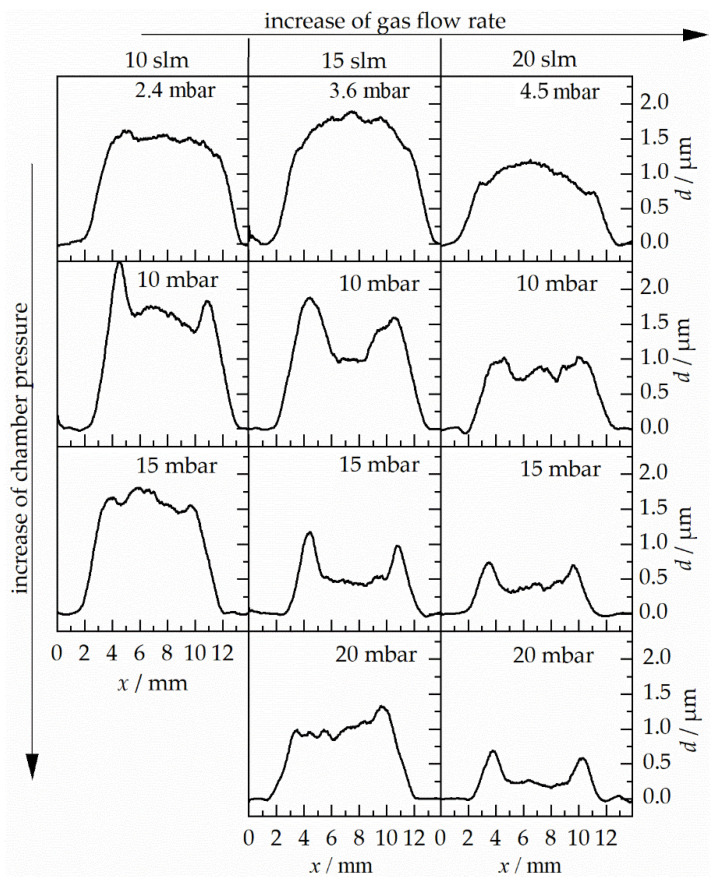
Film thickness profiles (film thickness (*d*) over film width (*x*)) of the as-deposited films prepared at different PAD parameter sets (variation of chamber pressure (*p*_c_) and gas flow rate ($\dot{V}$)

The surface profiles of the as-deposited films are plotted in Figure 8.

The thickest film was generated at a gas flow rate of 15 slm and the minimum possible chamber pressure (3.6 mbar). At a gas flow rate of 10 slm, the average film thickness was essentially the same for all chamber pressures, apart from the boundary pileups in the film deposited at 10 mbar. For flow rates of 15 slm (except at 20 mbar) and 20 slm, the film thickness decreases at increasing chamber pressures. Another decrease of the film thickness was observed as the gas flow rate was further increased (except 15 slm, 3.6 mbar).

The increase of the chamber pressure reduced the pressure ratio (*p*_i_/*p*_c_), the driving force for gas and particle acceleration. The particle velocity at impact should decrease with increasing chamber (back)pressure (*p*_c_). The interpretation of the images taken at increasing gas flow rates follows a similar line of argumentation. Increasing gas flow rates (i.e., increasing stagnation pressures) at constant chamber pressure shifts the Mach disk closer to the substrate (cf. Figure 7). Hence, the length of the supersonic section of the particle acceleration path increases, and therefore, the particle kinetic energy at impact on the substrate increases. Excessive high gas flow rates may cause particles to act increasingly abrasively, which would result in thinner deposit layers. In cold spray, this phenomenon is referred to as erosion regime, a decrease of deposition efficiency.

Coatings produced with parameter sets that yield similar shadow images (connected by dashed arrows in Figure 7), have qualitatively similar thickness profiles, but they are far from being identical. This is attributed to the different particle acceleration and resulting particle velocities. While the flow pattern seen in the shadow images only depends on the pressure ratio (*p*_i_/*p*_c_) and not on the absolute pressures, the drag acceleration of the particles is proportional to the pressure (*p*_i_) in the aerosol generator. At the same time, *p*_i_ is proportional to the total mass flow rate of the choked nozzle flow [28]. Therefore, particle acceleration is proportional to the gas flow rate at fixed pressure ratio. The evolution of the thickness profile and the total gas flow, both depend on the stagnation pressure in the aerosol generator. They correlate, while none is the cause of the other.

Therefore, similar flow patterns (i.e., identical pressure ratios) do by no means imply similar particle impact velocities on the substrate, let alone identical thickness profiles of the deposited films. The shadowgraph does not give any indication of the total pressures or densities in effect, nor of the actual particle acceleration or velocities. Flow imaging itself is not a tool for particle velocimetry, unless the stagnation pressure (*p*_i_) is taken into account and the flow field is gaged to actual gas densities. The images are utterly useful for putting the substrate in the right place, though. For instance, in order to gain maximal particle acceleration, the substrate may be purposely placed to shift the bow shock to the position of the Mach disk. All one needs to know is where the Mach disk is actually located. Such tricks have helped to improve coatings produced by high-pressure cold spray, too.

**Figure 9:**
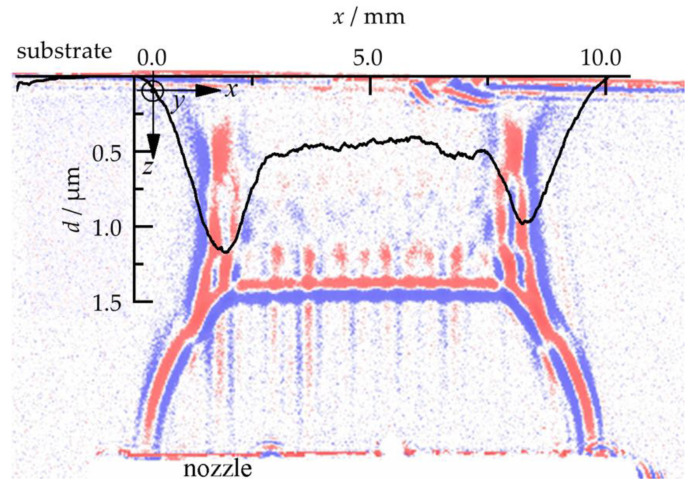
Film thickness profile (thickness (*d*) over width (*x*)) of an as-deposited film superposed with its corresponding shadow image (*x*-*z*-plane).

Nearly all thickness profiles that are generated at higher chamber pressures than the minimum value show peaks at the left and right ends of the deposition zone. This can be attributed to the lateral compression of the gas shown in Figure 7d–f,h,i,l. A more rigorous explanation is due to Figure 9. Here, a film thickness profile and its corresponding shadow image are superposed.

Particles leaving the nozzle at its left or right end gain a momentum towards the center plane as they interact with the intercepting shock. The compression waves have this direction and the density between the gas jet boundary and the intercepting shock (region D) is higher than in region A. A fraction of the particles is focused to these two spots on the substrate where the peaks form. More particles at one spot increase the possibility for film formation, because more particles with the right particle size, kinetic energy, crystallite size, and other required properties interact with the substrate. The local coating thickness depends on the local particle velocities, impact angles, and total impingement rate. All of these parameters are governed by the complex geometrical gas flow pattern with various zones of different particle acceleration potential, as seen in Equation (1). Thus, careful adjustment, or even design, of that gas flow is required in order to eventually obtain deposit films of uniform thickness. Shadowgraph imaging is recommended as a valuable diagnostic tool for optimization of the PAD gas flow.

## 4. Conclusions

The influence of the carrier gas flow rate and the chamber pressure on the formation of PAD-films was examined by correlating shadowgraph images of the gas flow between nozzle and substrate with as-deposited PAD-films. MgB_2_ particles were deposited on glass substrates. Higher chamber pressures lead, for this material, to a decrease in film thickness. Higher gas flow rates lead to a higher kinetic energy of the impacting particles. With constant chamber pressure and increasing gas flow rate, the impact energy of the particles increases, whereby the particles can exert an increasingly abrasive effect on the developing film coating. Material pileups at the edges of the film in *x*-*z*-plane can appear when the lateral compression of the gas flow increases due to higher chamber pressures. The existence of such thickness peaks indicates that the formation of the intercepting shock and boundary layer are crucial effects for the resulting uniformity of the film coating. The highest film thickness was generated at 15 slm. For the PAD-process, a low chamber pressure seems to be beneficial.

The shadow imaging system also gives important and valuable hints to the particle flow that is actually invisible. Shadowgraph imaging is an appreciated tool for validation of CFD simulations of the PAD process, as it allows estimation, e.g., of the true shape of gas streamlines and particle trajectories. Those can be compared to model calculations, like those by Zabihi Yeganeh et al. and Katanado et al. [20,21]. Moreover, shadowgraph imaging allows for purposeful location of the substrate in certain flow regions, as convenient, to obtain desired coating properties, e.g., uniform thickness. Altogether, it is certainly helpful when optimizing the PAD process parameter sets and nozzle shapes for the creation of possibly uniform coatings.

## Figures and Tables

**Table 1:** Parameter sets for flow imaging and film deposition.

Gas Flow Rate $\dot{V}/\mathrm{slm}$	Chamber (Back) Pressure/mbar	Number of Scans *
10	2.6	20
	10	
	15	
	20	
15	3.6	14
	10	
	15	
	20	
20	4.5	10
	10	
	15	
	20	

* parameter only in deposition experiments.

## Data Availability

The data presented in this study are available in the article.
